# Thermal Decomposition of Bio-Based Plastic Materials

**DOI:** 10.3390/molecules29133195

**Published:** 2024-07-05

**Authors:** Inés Oliver, Juan A. Conesa, Andres Fullana

**Affiliations:** 1 Institute of Chemical Process Engineering, University of Alicante, Ap. 99, 03080 Alicante, Spain; ines.oliver@ua.es (I.O.); andres.fullana@ua.es (A.F.); 2 Department of Chemical Engineering, University of Alicante, Ap. 99, 03080 Alicante, Spain

**Keywords:** bioplastic, pyrolysis, circular economy, thermoplastic starch (TPS), waste management

## Abstract

This research delves into a detailed exploration of the thermal decomposition behavior of bio-based polymers, specifically thermoplastic starch (TPS) and polylactic acid (PLA), under varying heating rates in a nitrogen atmosphere. This study employs thermogravimetry (TG) to investigate, providing comprehensive insights into the thermal stability of these eco-friendly polymers. In particular, the TPS kinetic model is examined, encompassing the decomposition of three distinct fractions. In contrast, PLA exhibits a simplified kinetic behavior requiring only a fraction described by a zero-order model. The kinetic study involves a systematic investigation into the individual contributions of key components within TPS, including starch, glycerin, and polyvinyl alcohol (PVA). This detailed analysis contributes to a comprehensive understanding of the thermal degradation process of TPS and PLA, enabling the optimization of processing conditions and the prediction of material behavior across varying thermal environments. Furthermore, the incorporation of different starch sources and calcium carbonate additives in TPS enhances our understanding of the polymer’s thermal stability, offering insights into potential applications in diverse industries.

## 1. Introduction

In the realm of sustainable materials, the exploration of bio-based plastics has become crucial for reducing our environmental footprint. Among the plethora of biodegradable polymers, polylactic acid (PLA), TPU (Thermoplastic Polyurethane), polyvinyl alcohol (PVA), starch-based polymers, and thermoplastic starch (TPS) have garnered significant attention. These materials, derived from renewable resources, hold promise as eco-friendly alternatives to conventional plastics.

Biopolymers, originating from renewable sources and intricately integrated into the biological framework, represent nature’s sophisticated response to the requirements for structural support, energy storage, and cellular communication. Positioned as sustainable alternatives to conventional polymers, biopolymers have attracted escalating interest due to their environmentally friendly attributes and potential applications across diverse industries. A key effort is understanding the thermal decomposition of biopolymers to see how they react to elevated temperature conditions.

This work focuses on the thermal decomposition of biopolymers, studying the behavior of biological macromolecules as they transform in response to thermal stimuli. Investigating these pathways is critical for optimizing the utilization of biopolymers in applications ranging from biomedical devices to sustainable packaging.

Exploring thermal decomposition becomes more insightful when examined from the perspective of kinetics. Kinetic models serve as essential tools, revealing the complex temporal changes in bio-based plastics as they react to heat, rather than being mere mathematical abstractions.

Examining reactions from a kinetic standpoint provides a pathway to understanding reaction rates and unraveling the complex interplay between temperature, time, and molecular structures. For PLA, this involves identifying the rate constants dictating its transition from a complex polymer matrix to simpler, more volatile compounds. Understanding the degradation kinetics of PLA allows us to go beyond static observations, gaining dynamic insights into the reactions that occur as temperature increases. In the case of PVA, starch-based polymers, and TPS, the kinetic lens brings forth a detailed understanding of the multi-step degradation processes.

PVA, starch-based polymers, and TPS present a diverse array of bio-based materials with unique properties and potential applications. Within the context of their thermal decomposition, our study extends to the development of kinetic models adapted to each material. These models serve as powerful tools for predicting degradation kinetics, offering insights into the factors influencing biodegradability and contributing to a more comprehensive understanding of their environmental impact. PLA has become a flagship bio-based polymer due to its biodegradability and versatile applications. It is widely used in 3D printing devices. Its low glass transition point, eco-friendly nature, and compatibility with various printers make it a popular choice. PLA’s ease of use and safety during printing contribute to its widespread adoption in both hobbyist and professional settings [1].

Beyond the laboratory bench, the kinetic models derived from the thermal decomposition of bio-based plastics have far-reaching implications for biodegradability and the overall life cycle of products. By integrating kinetic data into life cycle assessments, we bridge the gap between laboratory experiments and real-world applications. This holistic approach provides a deeper understanding of how thermal treatments influence the degradation kinetics and subsequent environmental fate of these materials, guiding decisions in waste management strategies and contributing to a circular economy.

TPS is typically produced through a process that involves blending starch with a plasticizer and, in some cases, additives. The starch, often derived from crops like corn, wheat, or potatoes, serves as the main biopolymer component. The plasticizer, which is commonly glycerol, is added to improve the flexibility and processability of the starch.

The process involves heating and mixing the starch and plasticizer, leading to gelatinization of the starch granules. This results in the formation of a homogeneous TPS matrix. After shaping, the material can be cooled and solidified to produce TPS. The final properties of TPS depend on factors such as the type of starch, plasticizer content, and any additional additives used in the formulation.

Some previous literature attempted to explain the thermal decomposition pathways of biopolymers. Guo et al. [2] demonstrated that PVA decomposition occurs in two stages, one peaking at 250 °C (523 K) and the second at 420 °C (693 K). In contrast, TPS exhibited a complex decomposition profile, with the maximum weight loss rate observed around 330 °C (603 K). During TPS decomposition, weight loss occurred at temperatures below 160 °C, attributed to water evolution and glycerol (plasticizer) decomposition.

In another study, Kaewtatip et al. [3] also identified the maximum rate of TPS decomposition at approximately 333 °C. Conversely, Karim et al. [4] reported that the maxima in the decomposition of glycerol–starch were centered at 305 °C. These authors emphasized the interaction between glycerol and starch in the film, noting that glycerol evaporation typically occurs at 180 °C, while starch decomposes at 340 °C [5].

Rahman et al. [6] prepared TPS blends with PVA and glycerol and found that increasing the PVA–glycerol content led to a decrease in both the onset and endpoint melting temperatures. The study also identified that the degradation of these blends occurs in three distinct phases: the vaporization of volatiles, the decomposition/dehydration and elimination of degradation products, and the formation of carbonaceous residues.

Regarding PVA decomposition, Sin et al. [7] prepared polymers using this alcohol in conjunction with cassava starch. The results indicated that neat cassava starch exhibited better thermal resistance than neat PVA, attributed to the presence of a cyclic hemiacetal structure in starch, conferring sustainability to thermal attacks. Blending these materials enhanced thermal stability, with the decomposition of pure PVA occurring at a maximum rate of 320 °C, whereas mixtures containing 50% alcohol with cassava starch shifted the decomposition to almost 400 °C (in runs performed at 20 K/min).

The global market for fillers moved more than EUR 8.5 billion in 2019, with calcium carbonate being one of the most used by the plastic industry due to its low cost, high availability, non-toxicity, and good thermal stability. It is expected that this market will grow annually by more than 5% until 2026 [8]. Studying the influence of this plastic additive in bioplastic formulations is deemed important. In the present work, key components of TPS, including starch, glycerin, and PVA, are subjected to systematic evaluation to delineate their individual contributions to the overall thermal degradation process. Furthermore, the incorporation of different starch sources and the addition of calcium carbonate in TPS serve as focal points for exploring the impact of these variables on the polymer’s thermal stability.

By undertaking this experimental effort, our goal is to offer valuable insights that aid in the optimization of processing conditions and enhance our ability to predict material behavior across diverse thermal environments. This research not only contributes to the growing body of knowledge in the field but also underscores the significance of experimental precision in advancing our understanding of the thermal characteristics of TPS and PLA.

This research investigates the impact of various starch sources and calcium carbonate additives on the thermal stability of TPS, comparing it to PLA and contributing new insights to the field. What distinguishes this study is its innovative approach of systematically evaluating biopolymer thermal decomposition using a kinetic model. By analyzing these factors, our aim is to improve the predictive accuracy of biopolymer behavior across diverse thermal environments, which is essential for advancing their practical applications.

## 2. Results

### 2.1. TPS Samples Using Different Starch

**Figure 1:**
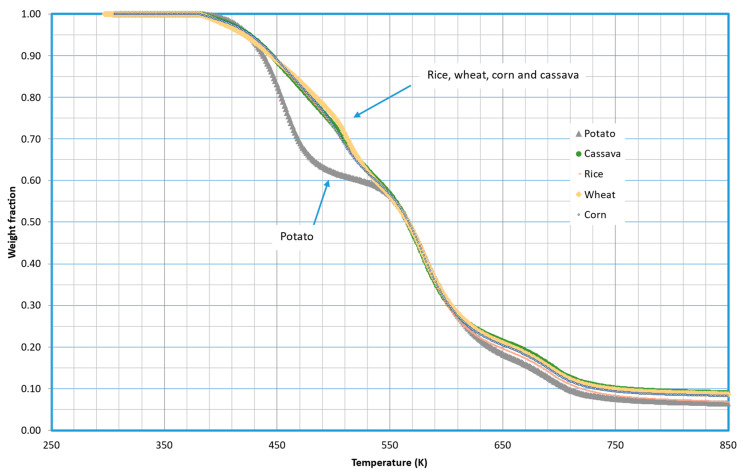
Comparison between the decomposition of different TPS samples using a variety of starch from different vegetables. All samples contain PVA, zinc stearate as a lubricant, and glycerin as a plasticizer in the same proportion. Curves were obtained at 5 K/min.

As mentioned previously, five different samples of TPS were tested using starch obtained from different vegetables. Figure 1 shows a comparison of the thermal decomposition in an inert atmosphere (pyrolytic runs) of these five materials at 5 K/min. The complete study at different heating rates is presented in the Supplementary Materials of this article (Figures S1–S5).

Significant variations in decomposition behavior were observed between TPS made from potato starch and all other tested materials. The initial observation reveals that potato-based TPS decomposes at a lower temperature, suggesting reduced temperature stability. Notably, the distinctions primarily manifest in the temperature range of 430 to 550 K (approximately 160 to 280 °C), beyond which the decomposition curves converge.

Although most starches typically contain 20–30% amylose and 70–80% amylopectin, these percentages vary based on the nature of the starch. Regarding amylose content, potato starch contains around 20%, wheat starch contains about 25%, corn starch contains between 55 and 75%, rice starch contains around 33%, and cassava starch contains about 20% [9,10]. Different amylose/amylopectin ratios impart various characteristics to TPS [11].

For the kinetic modeling of these decompositions, three fractions of materials were considered (*i* = 3). However, bearing in mind the form of the curves shown in Figure 1, a single group of parameters was considered for the decomposition of corn, wheat, rice, and cassava, which furthermore coincides with the final stages of the decomposition of potato-based TPS. In this sense, Table 1 shows the optimized values of the parameters. Note that the decomposition of Fractions 2 and 3 coincides for all five tested materials.

**Figure 2:**
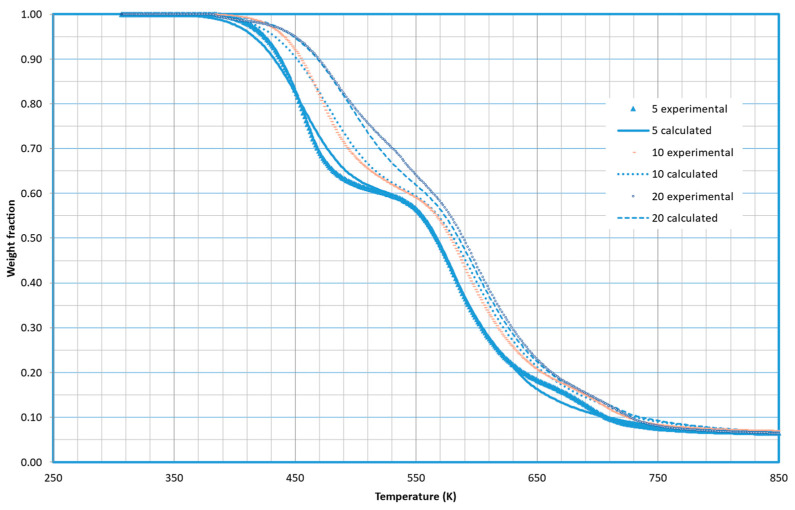
Fitting obtained for the decomposition of TPS based on a potato for the three different heating rates tested.

As an example of the quality of the fittings obtained, Figure 2 demonstrates the fitting of potato-based TPS at the three different heating rates tested in this work. The model effectively explains the experimental results.

The parameters obtained indicate that the samples contain three different materials from the thermal decomposition point of view. The first one accounts for almost 40% of the material (as s_10_ equals 0.3996), the second one accounts for 53.5%, and the rest are in a third fraction (almost 1%). The contribution of this last fraction to the weight is negligible.

**Figure 3:**
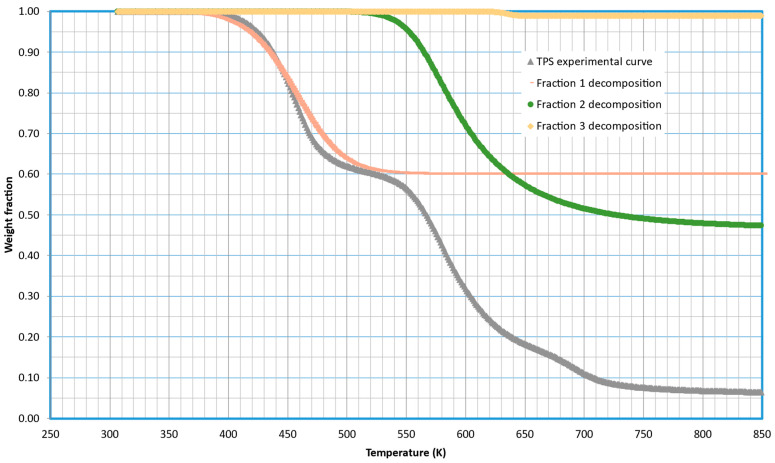
Details of the decomposition of the different fractions modeled for the thermal decomposition of TPS potato-based starch.

During the mathematical treatment of the kinetic model, it is possible to simulate the decomposition of each of these fractions separately. Figure 3 illustrates this simulation, displaying the decomposition and contribution (*s_i_*_0_) of each fraction to the global curve. The decomposition of the first fraction is centered around 450 K (at 5 K/min that is the heating rate shown in Figure 3), the second one is centered at 600 K, and the third one does not contribute appreciably to the total weight loss, although it is centered at 635 K.

**Figure 4:**
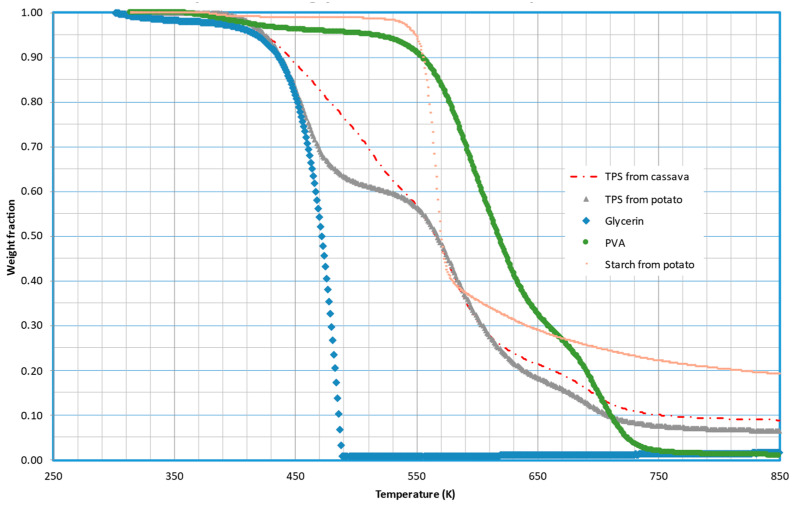
Thermal decomposition of pure starch from potato, glycerin, PVA, and TPS made using potato and cassava starch. Curves were obtained at 5 K/min.

To determine the possible composition of these fractions, runs were performed using the different components of the TPS separately. Potato starch, PVA, and glycerin were subjected to pyrolytic decomposition at three heating rates, representing the TPS composition. Figure 4 displays the results at 5 K/min, with the decomposition of TPS made from cassava starch included for comparison. It is evident that the initial loss of volatiles from TPS aligns closely with the decomposition of glycerin. The second peak in TPS decomposition can largely be attributed to starch decomposition, while PVA decomposition occurs over a broad temperature range (approximately 450–750 K), overlapping with the evolution of other fractions.

As previously mentioned, Sin et al. [7] observed a significant interaction between PVA and cassava starch. In our study, we also observed this interaction; however, it was not evident between PVA and potato starch. These results suggest that this interaction may not be as effective when using potato starch. In this sense, the interaction is of minor significance, and the sum of decompositions from different components essentially yields the decomposition of the processed material. Looking at Figure 4, it is evident that the sum of the decomposition of glycerin, PVA, and starch, in their respective proportions, yields a curve that is equivalent to the decomposition of potato TPS. However, the decomposition of cassava TPS could not be explained as a weighted sum of its components.

The decomposition of the components of the TPS was also tested for a kinetic study. Table 2 shows the results of the optimized parameters. Note that only two fractions (*i* = 2) were necessary to explain the decomposition of pure starch, PVA and glycerin. In all cases, three heating rates (5, 10, and 20 K/min) were simultaneously fitted. In the case of glycerin, almost only one fraction (s_10_ = 0.98) is able to explain the decomposition. Curiously, the decomposition of glycerin shows a zero-order kinetic behavior. This is typical for evaporation processes [12]. Glycerin, a trihydric alcohol, and a common component in various industries, exhibits a remarkable physical characteristic at room temperature. With a melting point of approximately 18 °C (291 K), glycerin defies conventional solid-state expectations, instead manifesting itself as a near liquid, underscoring its unique and versatile nature.

### 2.2. TPS with CaCO_3_ Added

Ref. [8] TPS-PVA blends were prepared with identical formulations, except for the weight percentage of calcium carbonate (CaCO_3_), to enable a comparison of their behavior.

**Figure 5:**
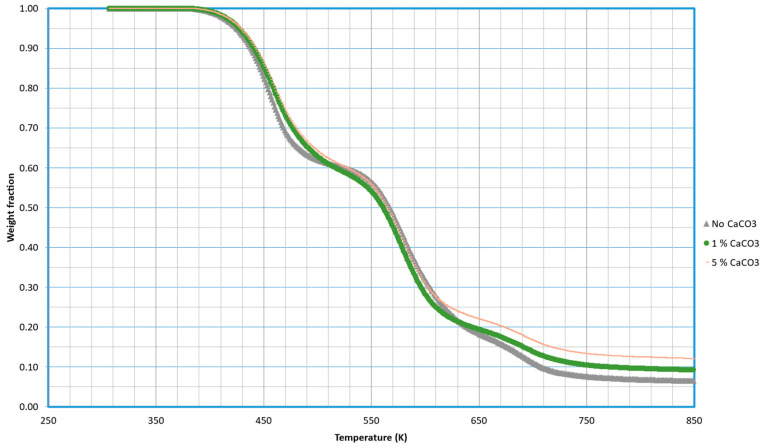
Thermal decomposition of TPS using starch from potato in the presence of different amounts of calcium carbonate. Curves were obtained at 5 K/min.

The analysis of the decomposition of the samples prepared with different CaCO_3_ content is presented in Figure 5. As in previous sections, only curves at 5 K/min are shown, although the complete set of curves can be found in the Supplementary Materials (Figures S6 and S7).

An interesting aspect of the thermal response of TPS emerges when investigating the influence of inert fillers under pyrolytic conditions. Contrary to expectations, the pyrolytic curves depicted in Figure 5 suggest that the presence of inert fillers has minimal impact on the decomposition kinetics of TPS. Under pyrolytic conditions, TPS appears to degrade independently of the inert fillers, maintaining its characteristic decomposition profile. This finding carries significant implications for the design and engineering of TPS-based materials, as it suggests a robust and predictable thermal behavior of TPS, regardless of the inert fillers incorporated.

### 2.3. PLA Decomposition

Another material tested in the present study is PLA. The relationship between TPS and PLA could be related to their applications and properties. Both are bioplastics derived from renewable sources, making them environmentally attractive compared to conventional petroleum-derived plastics. Additionally, both TPS and PLA are biodegradable and compostable under certain conditions, making them useful in applications where environmentally friendly degradation is required. From a research perspective, the relationship between TPS and PLA could focus on their compatibility as materials in a composite matrix or their combination to enhance certain properties of end products. For example, blends of TPS and PLA could be explored to achieve better mechanical strength, increased biodegradability, or improved barrier properties in biodegradable packaging.

**Figure 6:**
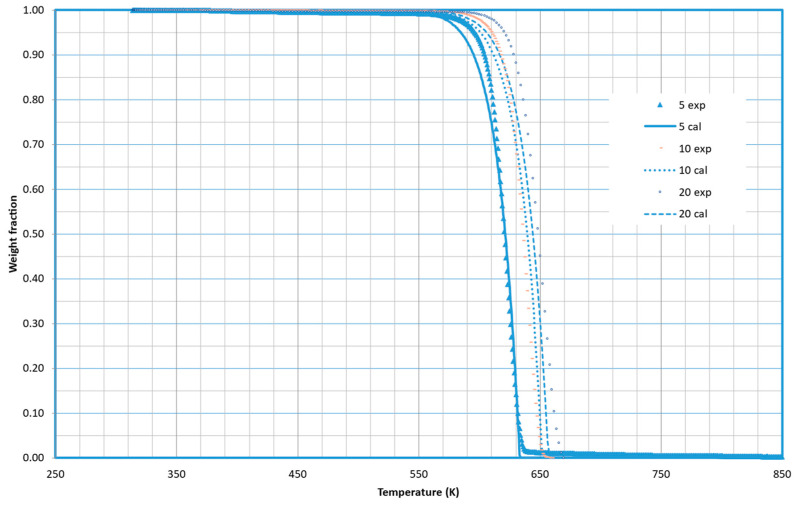
Thermal decomposition of PLA at three different heating rates under pyrolytic conditions. Experimental and calculated curves are shown.

Figure 6 shows the experimental and calculated weight fraction values at different heating rates. The optimized values of the kinetic constants reveal that it is only necessary to consider one fraction (*i* = 1) for the decomposition of PLA. The optimized values of the constants are shown in Table 3.

The thermal degradation of PLA, a biodegradable and renewable polymer, is marked by its simplicity and efficiency. Unlike some polymers that undergo multi-step decomposition, PLA exhibits a notable characteristic—its decomposition takes place in a singular, well-defined step [13]. This one-step degradation process is indicative of PLA’s inherent homogeneity and structural integrity. As PLA is subjected to elevated temperatures, it seamlessly undergoes depolymerization, transitioning from a complex macromolecular structure to simpler components without the need for intermediate stages. This unique attribute not only simplifies the understanding of PLA’s thermal behavior but also enhances its attractiveness in various applications, offering a streamlined approach to harnessing its eco-friendly properties in industries ranging from packaging to biomedical devices. The unequivocal single-step decomposition of PLA stands as a testament to its efficiency and lends itself to further exploration for sustainable material development.

It is worth noting that zero-order kinetics is found for PLA decomposition. The pyrolytic decomposition of PLA introduces an interesting aspect to its thermal behavior, as it unfolds in a liquid phase. Unlike traditional solid-state pyrolysis, PLA exhibits a distinctive transition into a liquid state during the decomposition process. This liquid-phase pyrolysis results from the specific chemical structure of PLA, which facilitates its transformation into a molten state under elevated temperatures. The remarkable characteristic of liquid-phase pyrolysis enhances the fluidity and mobility of PLA molecules during degradation, promoting efficient depolymerization. Understanding and harnessing this liquid-phase pyrolytic decomposition of PLA hold significant implications for optimizing processing conditions and tailoring end-product properties in various applications, ranging from the production of bio-based fuels to the design of advanced materials with tailored functionalities. This aspect of PLA’s pyrolysis opens new avenues for exploring and engineering sustainable materials with satisfactory performance characteristics.

## 3. Materials and Methods

### 3.1. Materials

In this work, a total of twelve samples were tested for pyrolytic decomposition. This study comprised samples of TPS using starch extracted from different vegetables (potato, corn, wheat, rice, and cassava) and potato-based TPS with varying amounts of added calcium carbonate. Additionally, samples other than TPS were tested, including PLA, and some components of the TPS polymers (potato starch, PVA, and glycerin, used as a plasticizer). The specific composition of the samples is shown in Table 4.

The starch film samples were prepared following the method outlined by Domene-Lopez et al. [14], with some modifications. Briefly, the raw materials mixture was processed using a HAAKE^TM^ PolyLab^TM^ QC Modular Torque Rheometer (ThermoFisher Scientific, Waltham, MA, USA). Processing involved subjecting the raw materials mixtures for the potato, corn, wheat, and rice samples, to a temperature of 110 °C for 10 min, with the initial 5 min at 50 rpm and the remaining time at 100 rpm. The cassava sample was obtained by applying 140 °C for 5 min, with the first minute at 50 rpm and the subsequent minutes at 100 rpm. Subsequently, the obtained blend was hot pressed at 160 °C for 10 min under a pressure of 7–10 tons, resulting in a 1.035 mm thick sheet.

Samples other than TPS were used as received. Starch extra pure powders (CAS: 9005-25-8) were purchased from ThermoFisher SCIENTIFIC, PVA (CAS: 9002-89-5 and MQ200), Zinc Stearate (CAS: 557-05-1 and MQ200), the latest one used as lubricant, was purchased from Sigma Aldrich (St. Louis, MO, USA), glycerin (CAS: 56-81-5 with ≥99%) was purchased from Fisher Chemicals (Waltham, MA, USA), and PLA (Total Corbion PLA) was purchased from TotalEnergies Corbion (Rayong, Thailand) with Luminity^®^ resin made from sugar cane.

### 3.2. Methods

Runs for the TG (thermogravimetric) analysis were conducted using a Mettler Toledo TGA/SDTA851e/SF/1100 Thermal Gravimetric Analyzer (Columbus, OH, USA). The decomposition temperatures were measured under dynamic conditions in a nitrogen atmosphere (pyrolytic runs) with a total flow rate of 100 mL min^−1^. Dynamic experiments were performed at heating rates of 5, 10, and 20 K min^−1^ from room temperature up to 1173 K. For each run, 6 ± 0.3 mg of the sample was used.

### 3.3. Kinetic Modeling

As different materials are studied in the present work, diverse kinetic models will be applied. The kinetic model proposed for the thermal decomposition of the different components considers each material to be composed of one to three independent parts (depending on the material), each following an independent reaction, as follows:

$$c_{Si0}\;Solid_{i}\overset{i-th\;reaction}{\to }\left(c_{Si0}-\upsilon_{i\infty }\right)Residue_{i}+\upsilon_{i\infty }Volatiles_{i}$$

with *i* = 1, 2, or 3. In the equation above, ‘*Solid_i_*’ refers to different fractions of the original material, ‘*Volatiles_i_*’ are the gases and condensable volatiles evolved in the corresponding reactions, and ‘*Residue_i_*’ is the possible residue formed in the decomposition of each ‘*Solid_i_*’.

The number of fractions for each material has been chosen through trial and error, determining the optimal number of fractions that best explain the experimental data with the objective function.

Each fraction has a yield coefficient (considered constant throughout the reaction) representing the maximum mass fraction obtainable by each reaction. Thus, ‘*v_i_*_∞_’ is the yield coefficient for the ‘*Volatiles_i_*’ and ‘(*c_Si_*_0_ − *v_i_*_∞_)’ is the yield coefficient for the ‘*Residue_i_*’. On the other hand, the sum of the initial mass fractions of the components ‘(*c_Si_*_0_)’ is exactly one minus the final mass fractions of the solid [15].

The conversion degree for each reaction is defined as the ratio between the mass fractions of solid reacted at any time ‘(*c_Si_*_0_ − *w_Si_*)’ and the corresponding initial fraction of this component as follows:

(1) $$\alpha_{i}=\frac{c_{Si0}-w_{Si}}{c_{Si0}},\;\;\;i=1,2,3$$

From the mass balance between products and reactants and the conversion degrees, the kinetic equations for the decomposition runs can be defined as follows:

(2) $$-\frac{d\left(\frac{w_{Si}}{c_{Si0}}\right)}{dt}=k_{i}{\left(\frac{w_{Si}}{c_{Si0}}\right)}^{n_{i}}\;\;or\;\;\frac{{d\alpha }_{i}}{dt}=k_{i}{\left(1-\alpha_{i}\right)}^{n_{i}}$$

with ‘*n_i_*’ is the reaction order and the kinetic constants following the Arrhenius equation as follows:

(3) $$k_{i}=k_{io}\cdot \exp\left(-\frac{E_{i}}{RT}\right)$$

For the calculation of the total mass remaining, a weighted sum is used as follows:

(4) $$-\frac{\left(d{w_{s}}^{cal}\right)}{dt}=\sum_{i}c_{Si0}\frac{{d\alpha }_{i}}{dt}\;\;\;and\;\;\;{w_{s}}^{cal}=1-\sum_{i}c_{Si0}\alpha_{i}\;$$

Considering the form of each curve (to be shown later), three different fractions (*i* = 3) were considered to explain the behavior of the different TPS samples, whereas two fractions (*i* = 2) were necessary to explain starch, PVA, and glycerin, and only one fraction (*i* = 1) for was necessary for PLA decomposition.

The optimization was performed by integrating the differential equations presented in the kinetic model using the finite differences method, considering and testing that the time intervals were small enough to make the integration errors negligible. The optimization method of the ‘Solver’ function in a Microsoft Excel^®^ (v. 16.14) spreadsheet was used to minimize the differences between the experimental and calculated mass fractions and their derivatives. The objective function (*O.F.*) to be minimized is as follows:

(5) $$O.F.={\sum_{m=1}^{M}\sum_{p=1}^{P}({w_{S}^{cal}}_{m,p}-{w_{S}^{exp}}_{m,p})}^{2}+factor{\sum_{m=1}^{M}\sum_{p=1}^{P}\left(\frac{{{dw}_{S}^{cal}}_{m,p}}{dt}-\frac{{{dw}_{S}^{exp}}_{m,p}}{dt}\right)}^{2}\;$$

where ‘*p*’ represents the experimental data at time ‘*t*’ in the experiment with a heating rate ‘*m*’. The value of *M* is the number of runs, and *P* is the number of points in each run. The value of the ‘factor’ was arbitrarily chosen to be 10^+3^ in order to give a similar contribution to the *O.F.* to the mass fraction differences and those of the derivatives [16]. Note that with this methodology, a unique set of kinetic constants is calculated from the experimental curves obtained at different heating rates [17,18,19], and it gives kinetic constants valid for the whole set of heating rates used.

As a result of the optimization, reaction orders different from unity are obtained. This fact can be due to the variation of the surface exposed to the decomposition as discussed elsewhere [20]. A reaction order greater than unity could indicate that there is a decrease in the surface exposed to the thermal decomposition, whereas a reaction order less than unity would imply that the surface exposed increases with the conversion.

## 4. Conclusions

The thermal decomposition behavior of TPS blends and PLA, which are both bio-based plastics, along with some of their components, such as pure potato starch, PVA, and glycerin, has been investigated. Kinetic models were developed for pyrolysis at three different heating rates: 5 K/min, 10 K/min, and 20 K/min.

The analysis of TPS samples using various starch sources revealed that TPS made from potato starch decomposes at lower temperatures compared to other types of starch. All TPS samples exhibited three fractions, with the contribution of the last one to the overall weight being negligible. Furthermore, the study of the decomposition of individual components revealed that the initial loss of volatile substances from TPS corresponds to the glycerin component, while the subsequent loss is attributed to starch decomposition.

Interestingly, the addition of CaCO_3_ as a filler to the TPS formulation did not result in a significant alteration of the decomposition profile. This suggests that the presence of CaCO_3_ has no substantial impact on the thermal degradation characteristics of TPS.

PLA demonstrated a unique, simple, and efficient one-step decomposition, indicating its homogeneity and structural integrity.

The findings presented in this article contribute valuable data for researchers, engineers, and industries striving to develop sustainable materials with enhanced thermal properties, acknowledging the distinct kinetic models governing the thermal decomposition of TPS and PLA.

## Figures and Tables

**Table 1:** Kinetic parameters optimized for the pyrolysis of TPS of different origins.

TPS from	Potato	Corn/Wheat/Rice/Cassava
log k_01_ (s^−1^)	4.89	3.51
E_1_ (kJ/mol)	65.04	55.70
n_1_	1.49	1.51
s_10_	0.3996	
log k_02_ (s^−1^)	13.88	
E_2_ (kJ/mol)	180.23	
n_2_	4.33	
s_20_	0.5358	
log k_03_ (s^−1^)	40.22	
E_3_ (kJ/mol)	552.82	
n_3_	1.16	
s_30_ (by difference)	0.0104	

**Table 2:** Optimized parameters for the pyrolysis of other materials.

	Starch from Potato	PVA	Glycerin
log k_01_ (s^−1^)	18.71	8.58	4.79
E_1_ (kJ/mol)	227.59	127.58	67.72
n_1_	0.46	2.40	0.01
s_10_	0.558	0.797	0.9800
log k_02_ (s^−1^)	89.27	24.52	6.73
E_2_ (kJ/mol)	991.52	362.16	58.03
n_2_	37.84	2.10	20.80
s_20_ (by difference)	0.296	0.203	0.0200

**Table 3:** Optimized parameters for the pyrolysis of PLA.

	PLA
log k_0_ (s^−1^)	12.44
E (kJ/mol)	179.32
n	0.00

**Table 4:** Composition of the samples in phr (parts per hundred of rubber).

	Origin	Starch	PVA	Glycerin	Zinc Stearate	CaCO_3_
S1	Potato	100	100	120	0.5 wt.%	-
S2	Potato	100	100	120	0.5 wt.%	1 wt.%
S3	Potato	100	100	120	0.5 wt.%	5 wt.%
S4	Potato *	100	100	120	0.5 wt.%	-
S5	Corn	100	100	120	0.5 wt.%	-
S6	Wheat	100	100	120	0.5 wt.%	-
S7	Rice	100	100	120	0.5 wt.%	-
S8	Cassava	100	100	120	0.5 wt.%	-
Other samples different from TPS						
S9	PLA					
S10	PVA					
S11	Starch from potato					
S12	Glycerin (plasticizer)					

* This sample was prepared using a different kind of PVA.

## Data Availability

Data are contained within the article and Supplementary Materials.

## Supplementary Materials

The following supporting information can be downloaded at https://www.mdpi.com/article/10.3390/molecules29133195/s1, Figure S1. Pyrolysis of TPS from potato starch at three heating rates. Figure S2. Pyrolysis of TPS from corn starch at three heating rates. Figure S3. Pyrolysis of TPS from wheat starch at three heating rates. Figure S4. Pyrolysis of TPS from rice starch at three heating rates. Figure S5. Pyrolysis of TPS from cassava starch at three heating rates. Figure S6. Pyrolysis of TPS from potato starch and 1% CaCO_3_ added at three heating rates. Figure S7. Pyrolysis of TPS from potato starch and 5% CaCO_3_ added at three heating rates.
