# Supplementary material for: Thermal Decomposition of Bio-Based Plastic Materials
Source: Molecules. 2024 Jul 5;29(13):3195. doi: 10.3390/molecules29133195 (PMC11243475; doi:10.3390/molecules29133195)
Supplement: Supplementary file 1 [file molecules-29-03195-s001.zip › molecules-3059060-supplementary.pdf]

Thermal Decomposition of Bio-Based Plastic Materials

Inés Oliver [1], Juan A. Conesa [1,2,*] and Andres Fullana [1,2]

[1] Institute of Chemical Process Engineering, University of Alicante, Ap. 99, 03080 Alicante, Spain; ines.oliver@ua.es (I.O.); andres.fullana@ua.es (A.F.)
[2] Department of Chemical Engineering, University of Alicante, Ap. 99, 03080 Alicante, Spain
* Correspondence: ja.conesa@ua.es

SUPPLEMENTARY MATERIAL

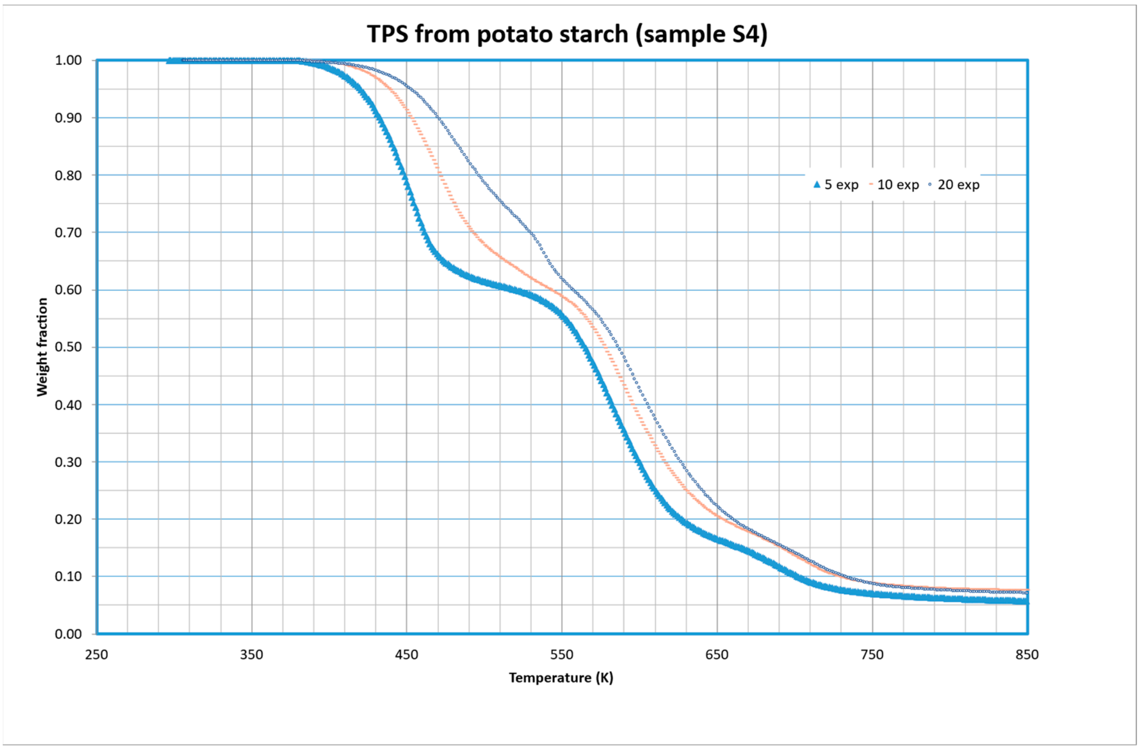

Figure S1. Pyrolysis of TPS from potato starch at three heating rates.

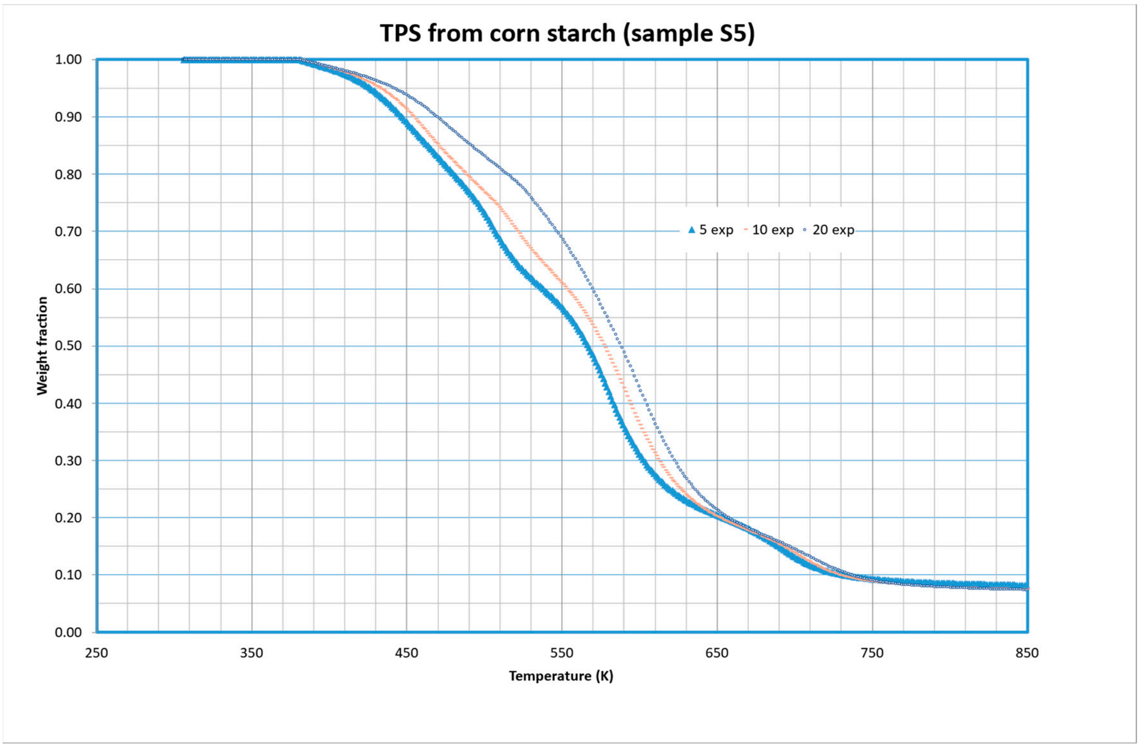

Figure S2. Pyrolysis of TPS from corn starch at three heating rates.

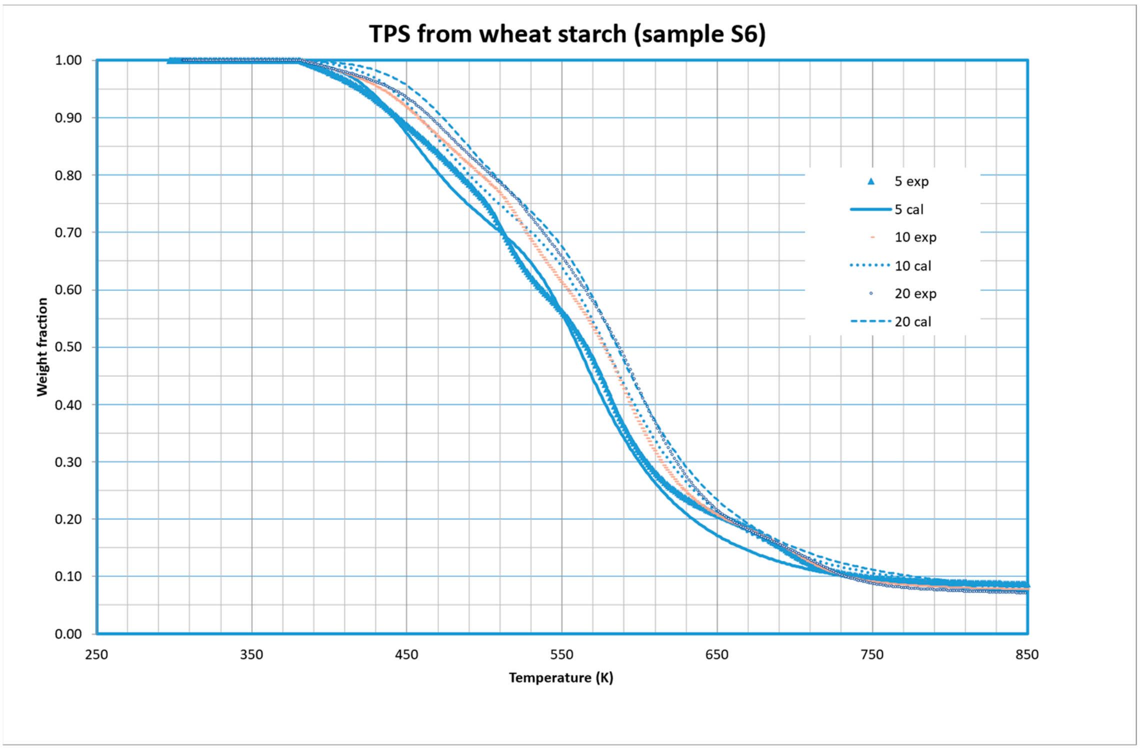

Figure S3. Pyrolysis of TPS from wheat starch at three heating rates.

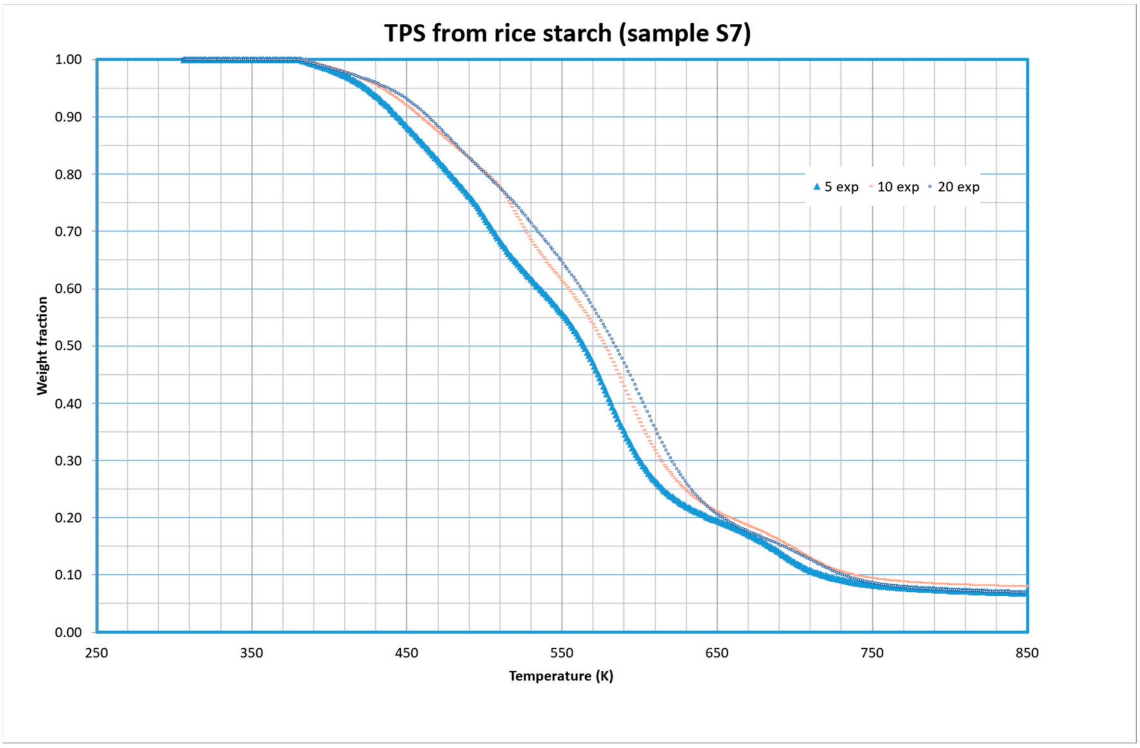

Figure S4. Pyrolysis of TPS from rice starch at three heating rates.

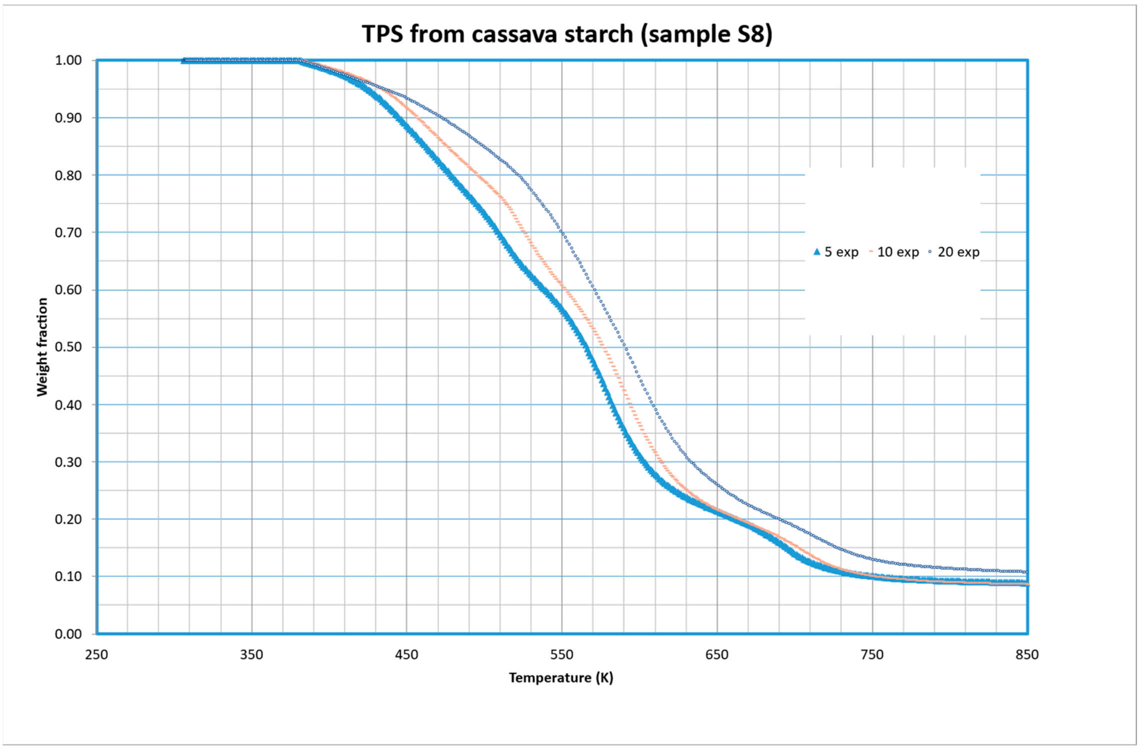

Figure S5. Pyrolysis of TPS from cassava starch at three heating rates.

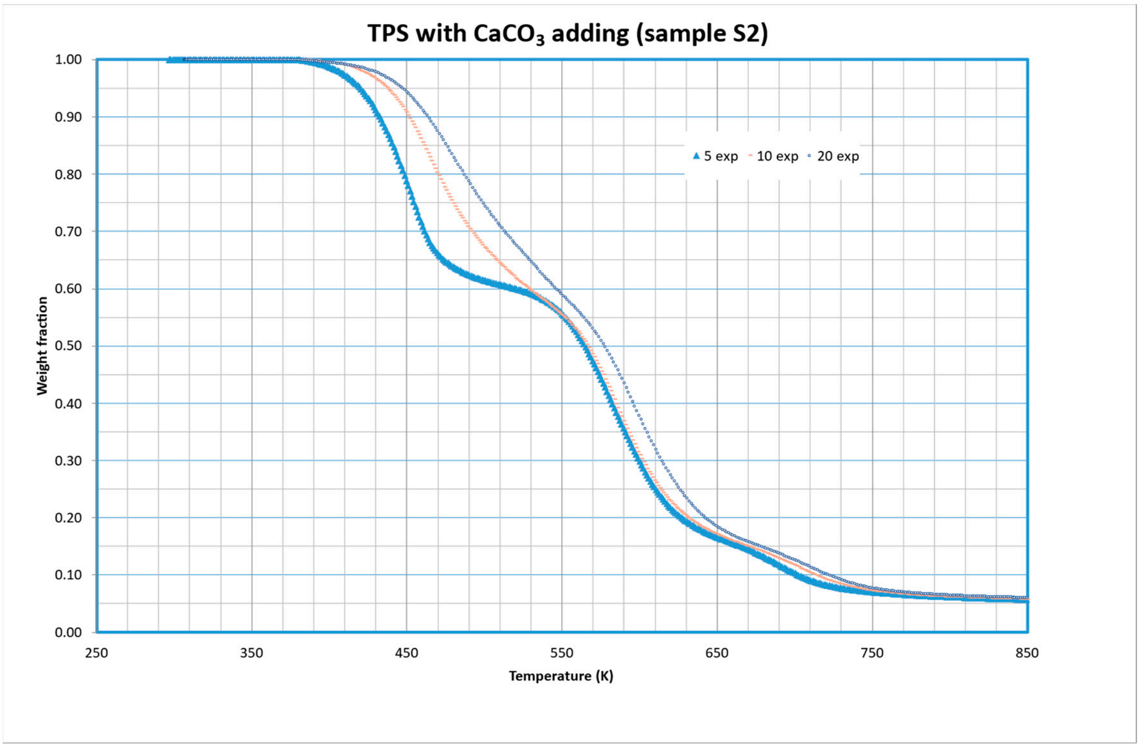

Figure S6. Pyrolysis of TPS from potato starch and 1 % $CaCO_3$ adding at three heating rates.

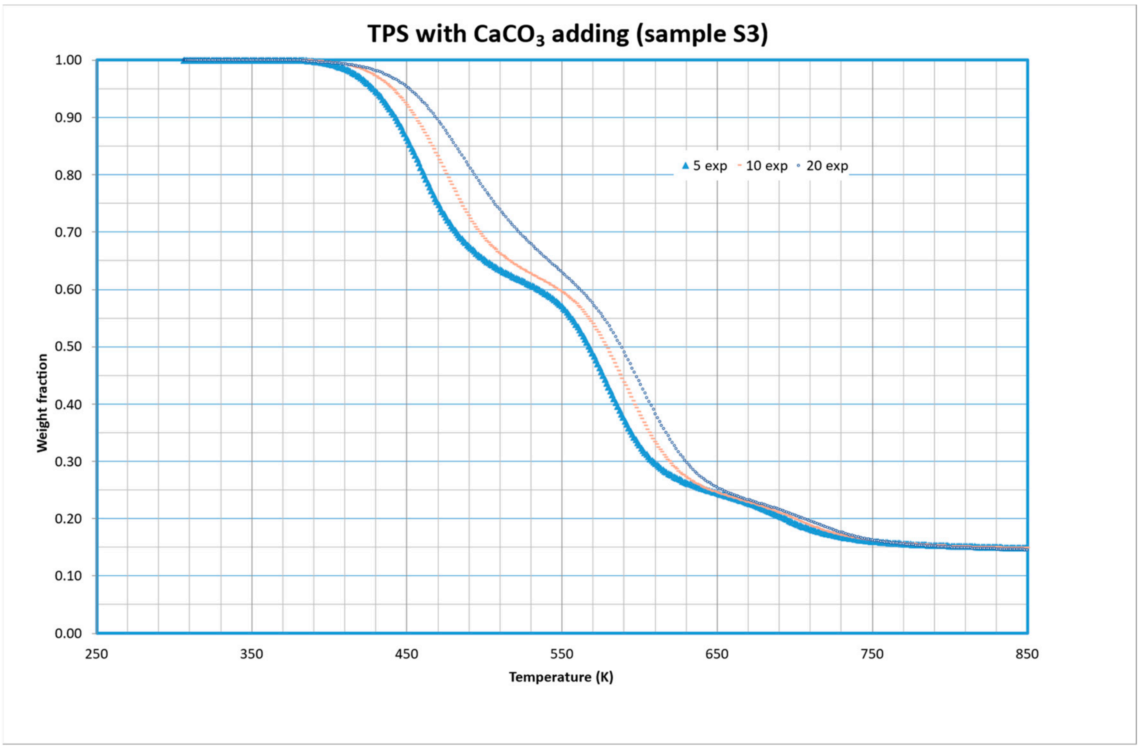

Figure S7. Pyrolysis of TPS from potato starch and 5 % $CaCO_3$ adding at three heating rates.
